# Supplementary material for: Shewanella is a putative producer of polyunsaturated fatty acids in the gut soil of the composting earthworm Eisenia fetida
Source: Appl Environ Microbiol. 2025 Jan 16;91(2):e02069-24. doi: 10.1128/aem.02069-24 (PMC11837533; doi:10.1128/aem.02069-24)

Figure S1. Amplicon rarefaction curves for rRNA sequences

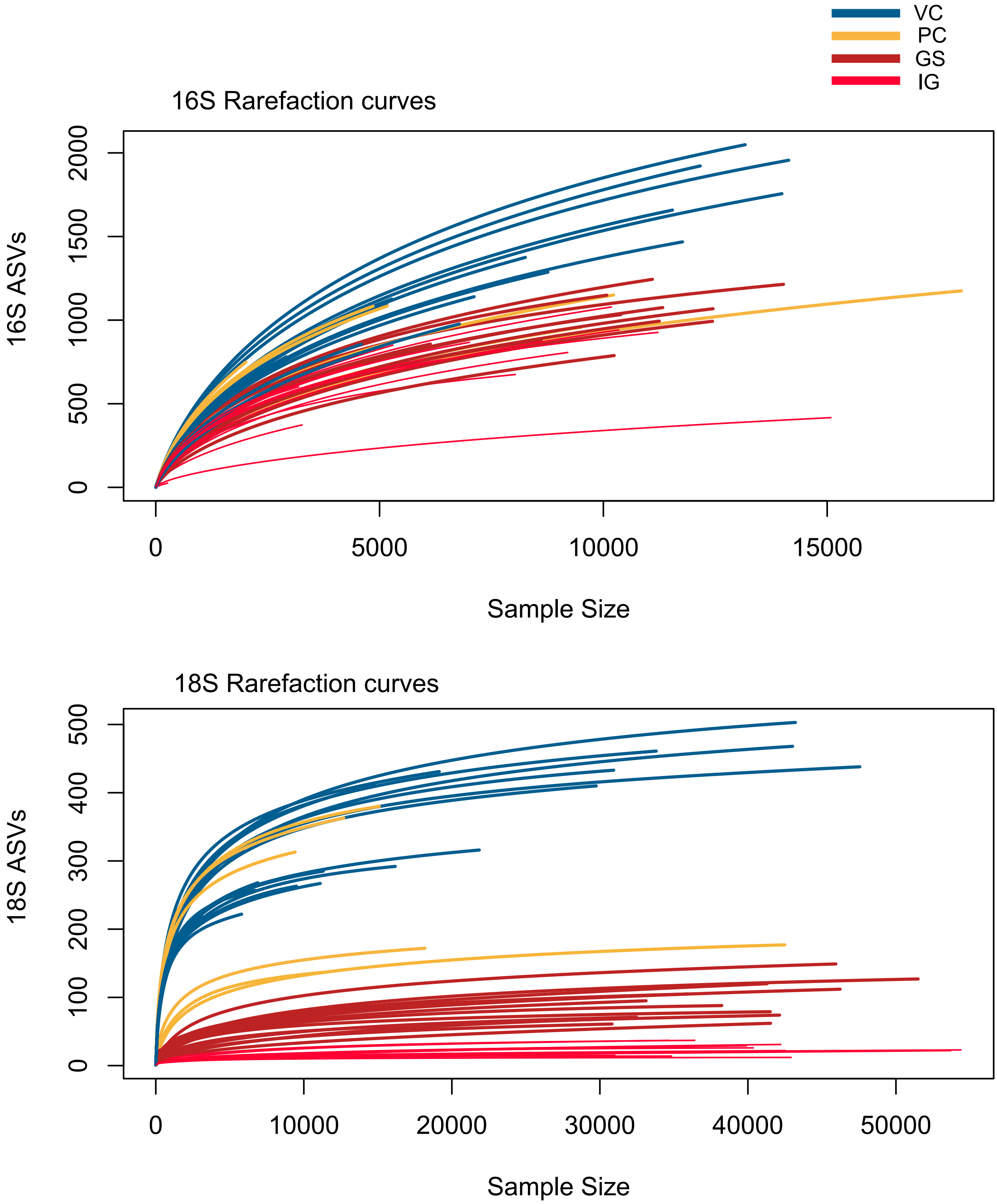

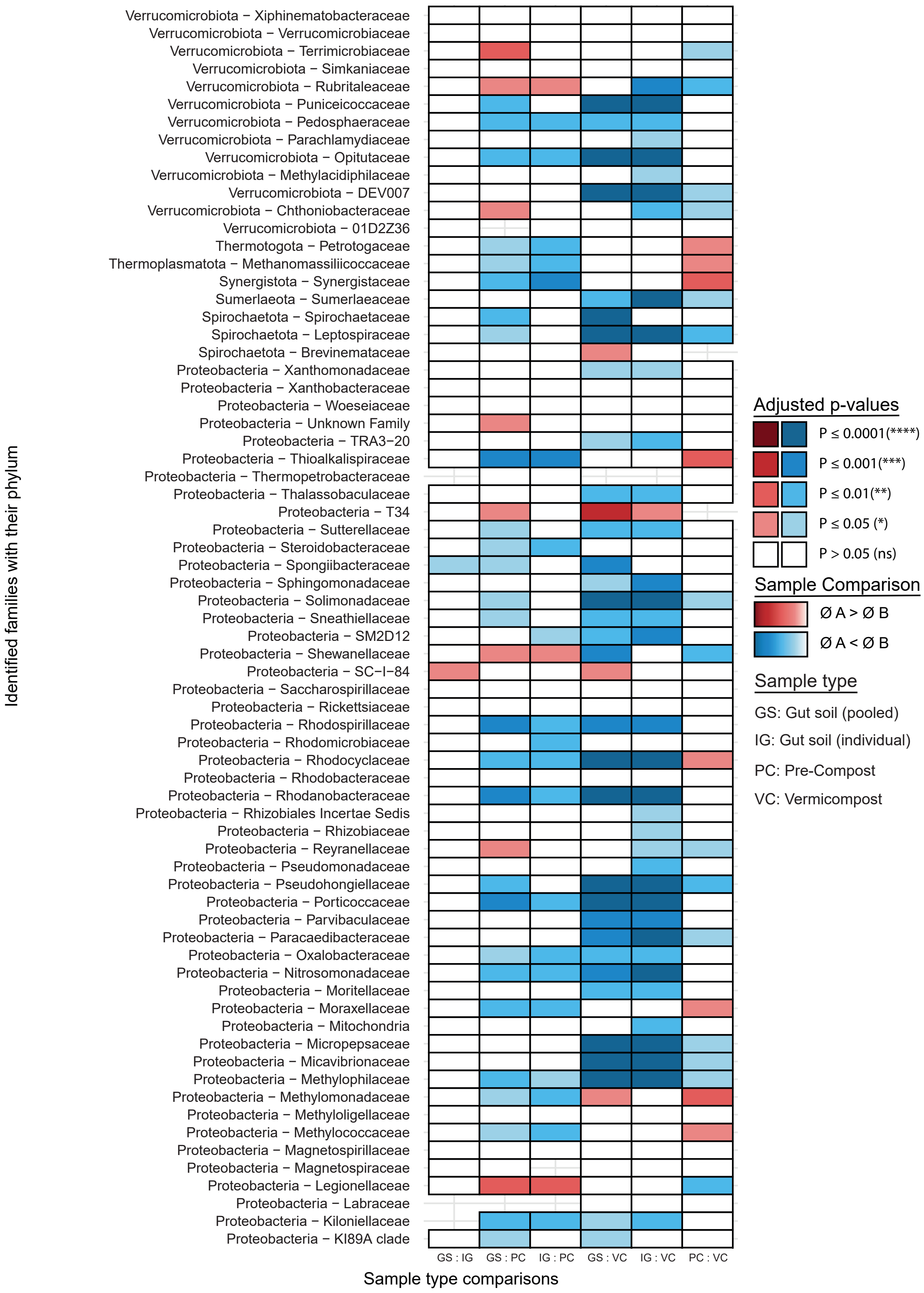
Verrucomicrobiota − Xiphinematobacteraceae
Verrucomicrobiota − Verrucomicrobiaceae
Verrucomicrobiota − Terrimicrobiaceae
Verrucomicrobiota − Simkaniaceae
Verrucomicrobiota − Rubritaleaceae
Verrucomicrobiota − Puniceicoccaceae
Verrucomicrobiota − Pedosphaeraceae
Verrucomicrobiota − Parachlamydiaceae
Verrucomicrobiota − Opitutaceae
Verrucomicrobiota − Methylacidiphilaceae
Verrucomicrobiota − DEV007
Verrucomicrobiota − Chthoniobacteraceae
Verrucomicrobiota − 01D2Z36
Thermotogota − Petrotogaceae
Thermoplasmatota − Methanomassiliicoccaceae
Synergistota − Synergistaceae
Sumerlaeota − Sumerlaeaceae
Spirochaetota − Spirochaetaceae
Spirochaetota − Leptospiraceae
Spirochaetota − Brevinemataceae
Proteobacteria − Xanthomonadaceae
Proteobacteria − Xanthobacteraceae
Proteobacteria − Woeseiaceae
Proteobacteria − Unknown Family
Proteobacteria − TRA3−20
Proteobacteria − Thioalkalispiraceae
Proteobacteria − Thermopetrobacteraceae
Proteobacteria − Thalassobaculaceae
Proteobacteria − T34
Proteobacteria − Sutterellaceae
Proteobacteria − Steroidobacteraceae
Proteobacteria − Spongiibacteraceae
Proteobacteria − Sphingomonadaceae
Proteobacteria − Solimonadaceae
Proteobacteria − Sneathiellaceae
Proteobacteria − SM2D12
Proteobacteria − Shewanellaceae
Proteobacteria − SC−I−84
Proteobacteria − Saccharospirillaceae
Proteobacteria − Rickettsiaceae
Proteobacteria − Rhodospirillaceae
Proteobacteria − Rhodomicrobiaceae
Proteobacteria − Rhodocyclaceae
Proteobacteria − Rhodobacteraceae
Proteobacteria − Rhodanobacteraceae
Proteobacteria − Rhizobiales Incertae Sedis
Proteobacteria − Rhizobiaceae
Proteobacteria − Reyranellaceae
Proteobacteria − Pseudomonadaceae
Proteobacteria − Pseudohongiellaceae
Proteobacteria − Porticoccaceae
Proteobacteria − Parvibaculaceae
Proteobacteria − Paracaedibacteraceae
Proteobacteria − Oxalobacteraceae
Proteobacteria − Nitrosomonadaceae
Proteobacteria − Moritellaceae
Proteobacteria − Moraxellaceae
Proteobacteria − Mitochondria
Proteobacteria − Micropepsaceae
Proteobacteria − Micavibrionaceae
Proteobacteria − Methylophilaceae
Proteobacteria − Methylomonadaceae
Proteobacteria − Methyloligellaceae
Proteobacteria − Methylococcaceae
Proteobacteria − Magnetospirillaceae
Proteobacteria − Magnetospiraceae
Proteobacteria − Legionellaceae
Proteobacteria − Labraceae
Proteobacteria − Kiloniellaceae
Proteobacteria − KI89A clade
Identified families with their phylum
GS : IG
GS : PC
IG : PC
GS : VC
IG : VC
PC : VC
Sample type comparisons
Adjusted p-values
P ≤ 0.0001(****)
P ≤ 0.001(***)
P ≤ 0.01(**)
P ≤ 0.05 (*)
P > 0.05 (ns)
Sample Comparison
Ø A > Ø B
Ø A < Ø B
Sample type
GS: Gut soil (pooled)
IG: Gut soil (individual)
PC: Pre-Compost
VC: Vermicompost

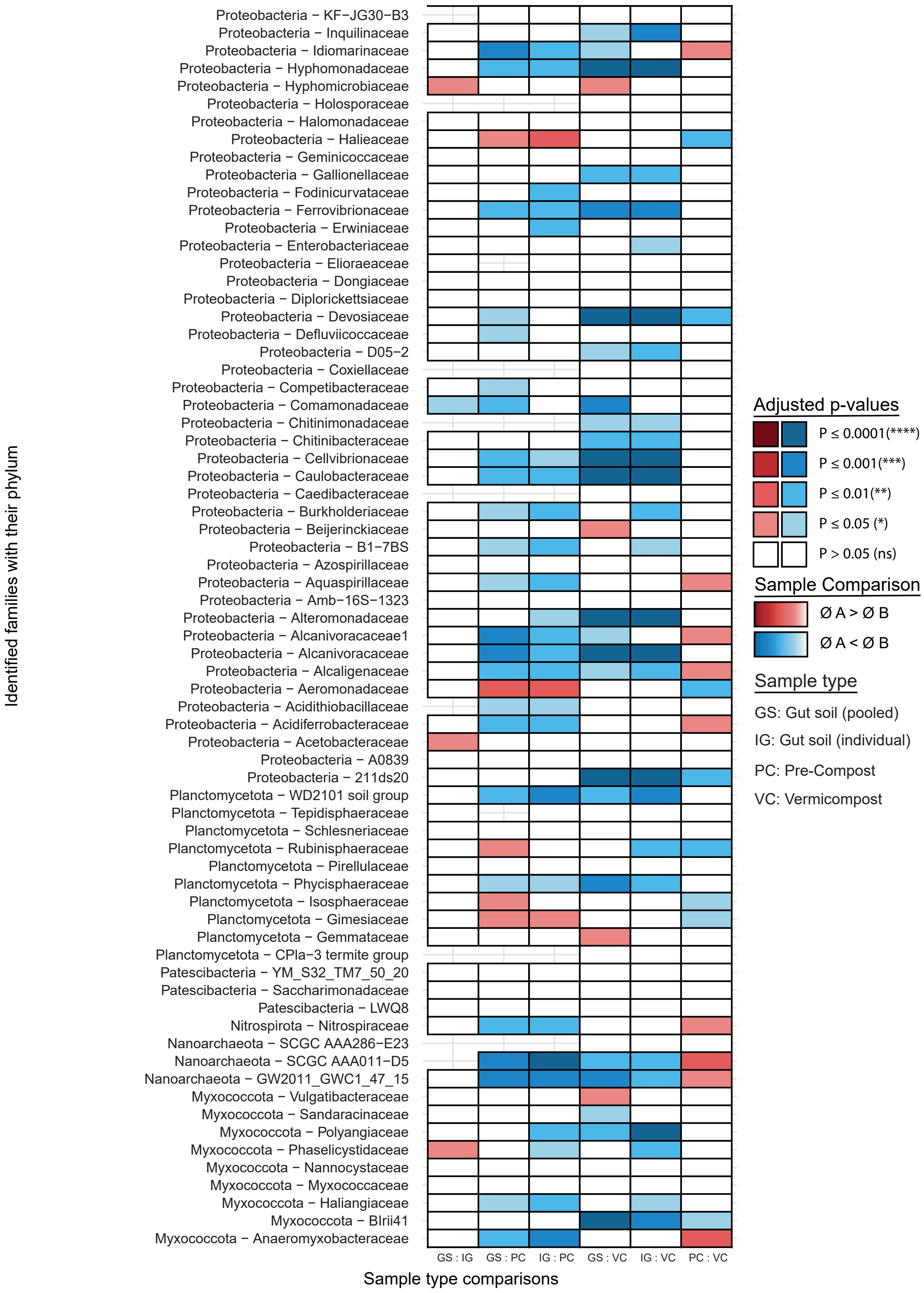
Proteobacteria − KF−JG30−B3
Proteobacteria − Inquilinaceae
Proteobacteria − Idiomarinaceae
Proteobacteria − Hyphomonadaceae
Proteobacteria − Hyphomicrobiaceae
Proteobacteria − Holosporaceae
Proteobacteria − Halomonadaceae
Proteobacteria − Halieaceae
Proteobacteria − Geminicoccaceae
Proteobacteria − Gallionellaceae
Proteobacteria − Fodinicurvataceae
Proteobacteria − Ferrovibrionaceae
Proteobacteria − Erwiniaceae
Proteobacteria − Enterobacteriaceae
Proteobacteria − Elioraeaceae
Proteobacteria − Dongiaceae
Proteobacteria − Diplorickettsiaceae
Proteobacteria − Devosiaceae
Proteobacteria − Defluviicoccaceae
Proteobacteria − D05−2
Proteobacteria − Coxiellaceae
Proteobacteria − Competibacteraceae
Proteobacteria − Comamonadaceae
Proteobacteria − Chitinimonadaceae
Proteobacteria − Chitinibacteraceae
Proteobacteria − Cellvibrionaceae
Proteobacteria − Caulobacteraceae
Proteobacteria − Caedibacteraceae
Proteobacteria − Burkholderiaceae
Proteobacteria − Beijerinckiaceae
Proteobacteria − B1−7BS
Proteobacteria − Azospirillaceae
Proteobacteria − Aquaspirillaceae
Proteobacteria − Amb−16S−1323
Proteobacteria − Alteromonadaceae
Proteobacteria − Alcanivoracaceae1
Proteobacteria − Alcanivoracaceae
Proteobacteria − Alcaligenaceae
Proteobacteria − Aeromonadaceae
Proteobacteria − Acidithiobacillaceae
Proteobacteria − Acidiferrobacteraceae
Proteobacteria − Acetobacteraceae
Proteobacteria − A0839
Proteobacteria − 211ds20
Planctomycetota − WD2101 soil group
Planctomycetota − Tepidisphaeraceae
Planctomycetota − Schlesneriaceae
Planctomycetota − Rubinisphaeraceae
Planctomycetota − Pirellulaceae
Planctomycetota − Phycisphaeraceae
Planctomycetota − Isosphaeraceae
Planctomycetota − Gimesiaceae
Planctomycetota − Gemmataceae
Planctomycetota − CPla−3 termite group
Patescibacteria − YM_S32_TM7_50_20
Patescibacteria − Saccharimonadaceae
Patescibacteria − LWQ8
Nitrospirota − Nitrospiraceae
Nanoarchaeota − SCGC AAA286−E23
Nanoarchaeota − SCGC AAA011−D5
Nanoarchaeota − GW2011_GWC1_47_15
Myxococcota − Vulgatibacteraceae
Myxococcota − Sandaracinaceae
Myxococcota − Polyangiaceae
Myxococcota − Phaselicystidaceae
Myxococcota − Nannocystaceae
Myxococcota − Myxococcaceae
Myxococcota − Haliangiaceae
Myxococcota − BIrii41
Myxococcota − Anaeromyxobacteraceae
Identified families with their phylum
GS : IG
GS : PC
IG : PC
GS : VC
IG : VC
PC : VC
Sample type comparisons
Adjusted p-values
P ≤ 0.0001(****)
P ≤ 0.001(***)
P ≤ 0.01(**)
P ≤ 0.05 (*)
P > 0.05 (ns)
Sample Comparison
Ø A > Ø B
Ø A < Ø B
Sample type
GS: Gut soil (pooled)
IG: Gut soil (individual)
PC: Pre-Compost
VC: Vermicompost

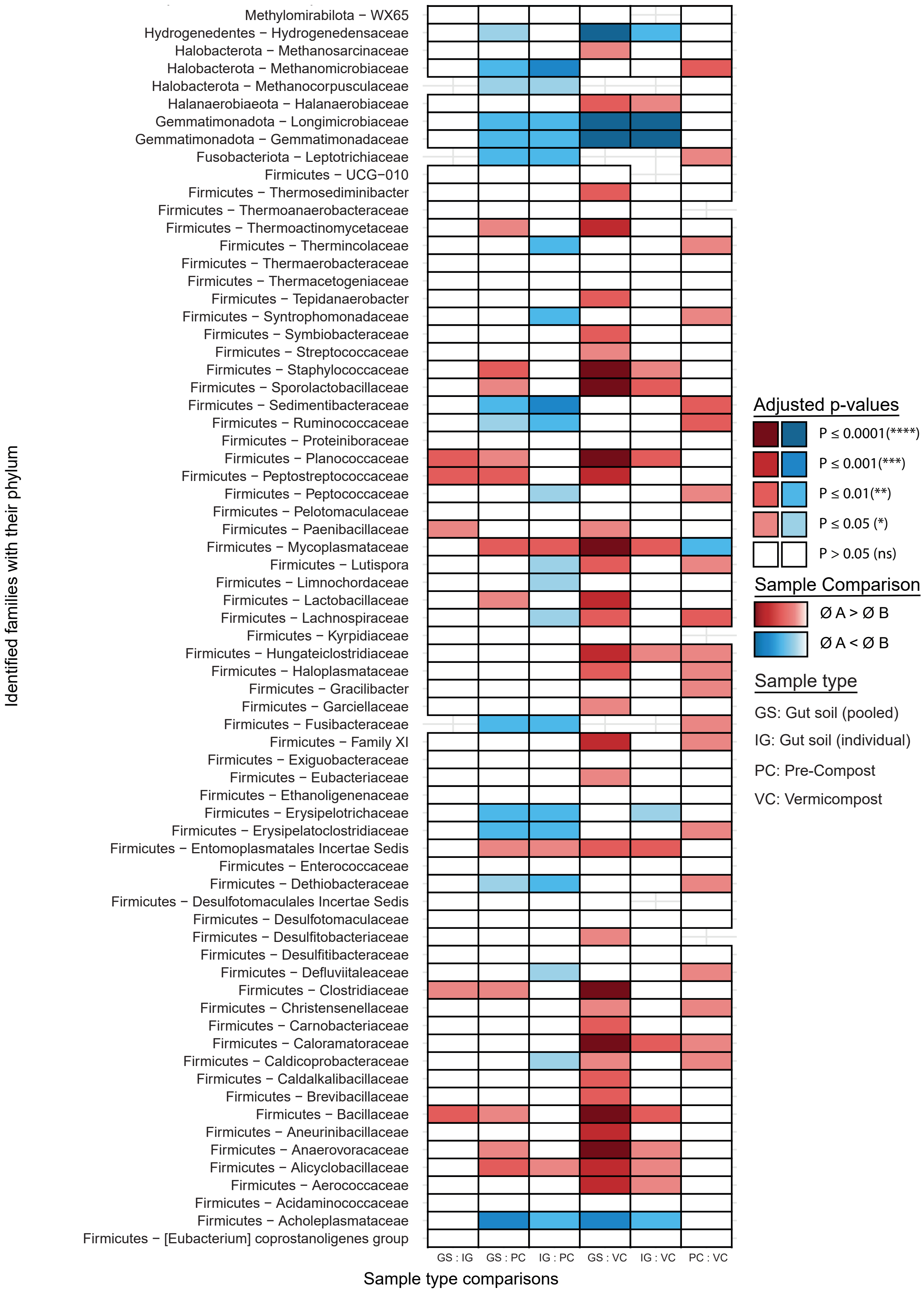
Identified families with their phylum
Methylomirabilota − WX65
Hydrogenedentes − Hydrogenedensaceae
Halobacterota − Methanosarcinaceae
Halobacterota − Methanomicrobiaceae
Halobacterota − Methanocorpusculaceae
Halanaerobiaeota − Halanaerobiaceae
Gemmatimonadota − Longimicrobiaceae
Gemmatimonadota − Gemmatimonadaceae
Fusobacteriota − Leptotrichiaceae
Firmicutes − UCG−010
Firmicutes − Thermosediminibacter
Firmicutes − Thermoanaerobacteraceae
Firmicutes − Thermoactinomycetaceae
Firmicutes − Thermincolaceae
Firmicutes − Thermaerobacteraceae
Firmicutes − Thermacetogeniaceae
Firmicutes − Tepidanaerobacter
Firmicutes − Syntrophomonadaceae
Firmicutes − Symbiobacteraceae
Firmicutes − Streptococcaceae
Firmicutes − Staphylococcaceae
Firmicutes − Sporolactobacillaceae
Firmicutes − Sedimentibacteraceae
Firmicutes − Ruminococcaceae
Firmicutes − Proteiniboraceae
Firmicutes − Planococcaceae
Firmicutes − Peptostreptococcaceae
Firmicutes − Peptococcaceae
Firmicutes − Pelotomaculaceae
Firmicutes − Paenibacillaceae
Firmicutes − Mycoplasmataceae
Firmicutes − Lutispora
Firmicutes − Limnochordaceae
Firmicutes − Lactobacillaceae
Firmicutes − Lachnospiraceae
Firmicutes − Kyrpidiaceae
Firmicutes − Hungateiclostridiaceae
Firmicutes − Haloplasmataceae
Firmicutes − Gracilibacter
Firmicutes − Garciellaceae
Firmicutes − Fusibacteraceae
Firmicutes − Family XI
Firmicutes − Exiguobacteraceae
Firmicutes − Eubacteriaceae
Firmicutes − Ethanoligenenaceae
Firmicutes − Erysipelotrichaceae
Firmicutes − Erysipelatoclostridiaceae
Firmicutes − Entomoplasmatales Incertae Sedis
Firmicutes − Enterococcaceae
Firmicutes − Dethiobacteraceae
Firmicutes − Desulfotomaculales Incertae Sedis
Firmicutes − Desulfotomaculaceae
Firmicutes − Desulfitobacteriaceae
Firmicutes − Desulfitibacteraceae
Firmicutes − Defluviitaleaceae
Firmicutes − Clostridiaceae
Firmicutes − Christensenellaceae
Firmicutes − Carnobacteriaceae
Firmicutes − Caloramatoraceae
Firmicutes − Caldicoprobacteraceae
Firmicutes − Caldalkalibacillaceae
Firmicutes − Brevibacillaceae
Firmicutes − Bacillaceae
Firmicutes − Aneurinibacillaceae
Firmicutes − Anaerovoracaceae
Firmicutes − Alicyclobacillaceae
Firmicutes − Aerococcaceae
Firmicutes − Acidaminococcaceae
Firmicutes − Acholeplasmataceae
Firmicutes − [Eubacterium] coprostanoligenes group
GS : IG
GS : PC
IG : PC
GS : VC
IG : VC
PC : VC
Sample type comparisons
Adjusted p-values
P ≤ 0.0001(****)
P ≤ 0.001(***)
P ≤ 0.01(**)
P ≤ 0.05 (*)
P > 0.05 (ns)
Sample Comparison
Ø A > Ø B
Ø A < Ø B
Sample type
GS: Gut soil (pooled)
IG: Gut soil (individual)
PC: Pre-Compost
VC: Vermicompost

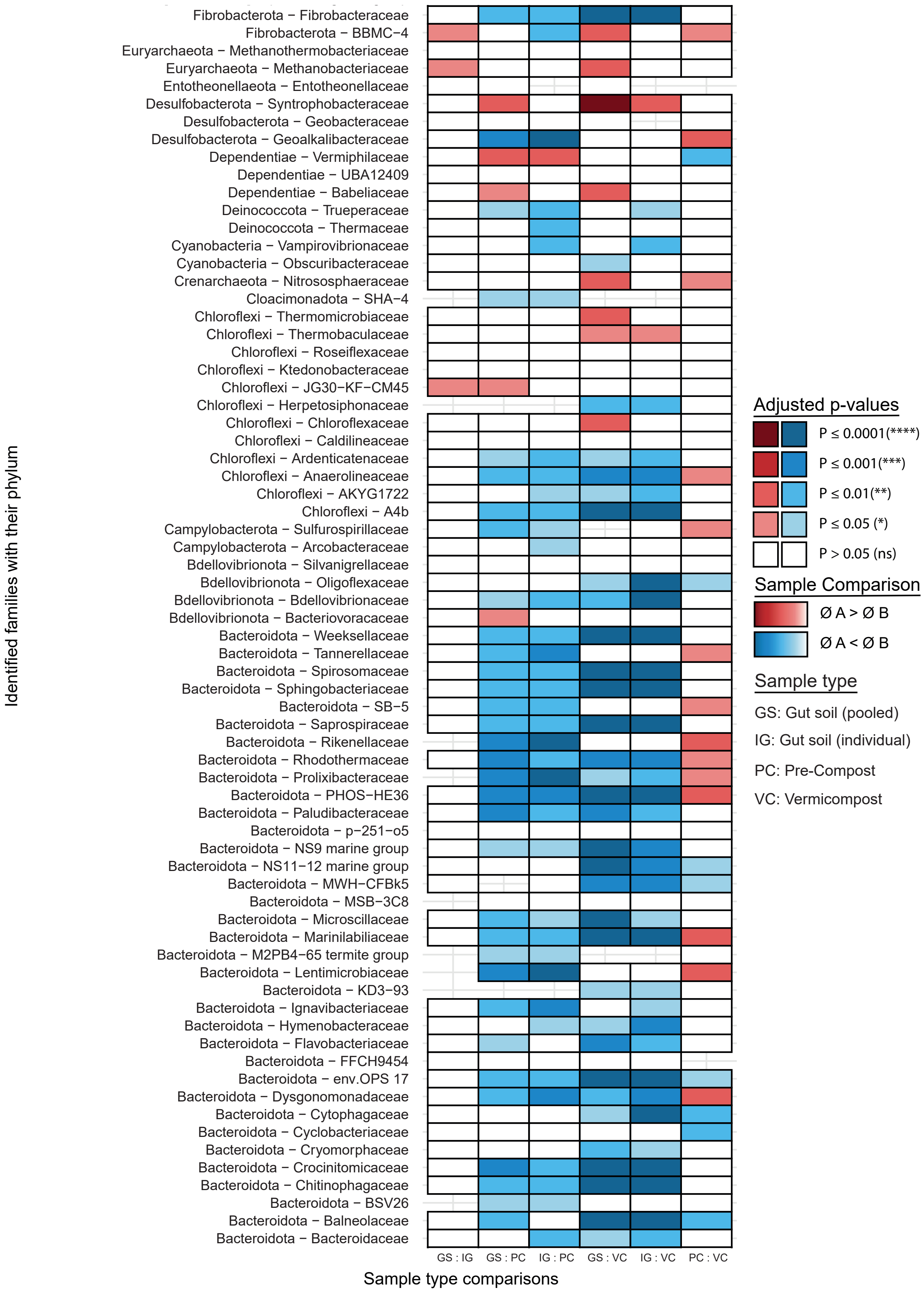
Identified families with their phylum
Fibrobacterota − Fibrobacteraceae
Fibrobacterota − BBMC−4
Euryarchaeota − Methanothermobacteriaceae
Euryarchaeota − Methanobacteriaceae
Entotheonellaeota − Entotheonellaceae
Desulfobacterota − Syntrophobacteraceae
Desulfobacterota − Geobacteraceae
Desulfobacterota − Geoalkalibacteraceae
Dependentiae − Vermiphilaceae
Dependentiae − UBA12409
Dependentiae − Babeliaceae
Deinococcota − Trueperaceae
Deinococcota − Thermaceae
Cyanobacteria − Vampirovibrionaceae
Cyanobacteria − Obscuribacteraceae
Crenarchaeota − Nitrososphaeraceae
Cloacimonadota − SHA−4
Chloroflexi − Thermomicrobiaceae
Chloroflexi − Thermobaculaceae
Chloroflexi − Roseiflexaceae
Chloroflexi − Ktedonobacteraceae
Chloroflexi − JG30−KF−CM45
Chloroflexi − Herpetosiphonaceae
Chloroflexi − Chloroflexaceae
Chloroflexi − Caldilineaceae
Chloroflexi − Ardenticatenaceae
Chloroflexi − Anaerolineaceae
Chloroflexi − AKYG1722
Chloroflexi − A4b
Campylobacterota − Sulfurospirillaceae
Campylobacterota − Arcobacteraceae
Bdellovibrionota − Silvanigrellaceae
Bdellovibrionota − Oligoflexaceae
Bdellovibrionota − Bdellovibrionaceae
Bdellovibrionota − Bacteriovoracaceae
Bacteroidota − Weeksellaceae
Bacteroidota − Tannerellaceae
Bacteroidota − Spirosomaceae
Bacteroidota − Sphingobacteriaceae
Bacteroidota − SB−5
Bacteroidota − Saprospiraceae
Bacteroidota − Rikenellaceae
Bacteroidota − Rhodothermaceae
Bacteroidota − Prolixibacteraceae
Bacteroidota − PHOS−HE36
Bacteroidota − Paludibacteraceae
Bacteroidota − p−251−o5
Bacteroidota − NS9 marine group
Bacteroidota − NS11−12 marine group
Bacteroidota − MWH−CFBk5
Bacteroidota − MSB−3C8
Bacteroidota − Microscillaceae
Bacteroidota − Marinilabiliaceae
Bacteroidota − M2PB4−65 termite group
Bacteroidota − Lentimicrobiaceae
Bacteroidota − KD3−93
Bacteroidota − Ignavibacteriaceae
Bacteroidota − Hymenobacteraceae
Bacteroidota − Flavobacteriaceae
Bacteroidota − FFCH9454
Bacteroidota − env.OPS 17
Bacteroidota − Dysgonomonadaceae
Bacteroidota − Cytophagaceae
Bacteroidota − Cyclobacteriaceae
Bacteroidota − Cryomorphaceae
Bacteroidota − Crocinitomicaceae
Bacteroidota − Chitinophagaceae
Bacteroidota − BSV26
Bacteroidota − Balneolaceae
Bacteroidota − Bacteroidaceae
GS : IG
GS : PC
IG : PC
GS : VC
IG : VC
PC : VC
Sample type comparisons
Adjusted p-values
P ≤ 0.0001(****)
P ≤ 0.001(***)
P ≤ 0.01(**)
P ≤ 0.05 (*)
P > 0.05 (ns)
Sample Comparison
Ø A > Ø B
Ø A < Ø B
Sample type
GS: Gut soil (pooled)
IG: Gut soil (individual)
PC: Pre-Compost
VC: Vermicompost

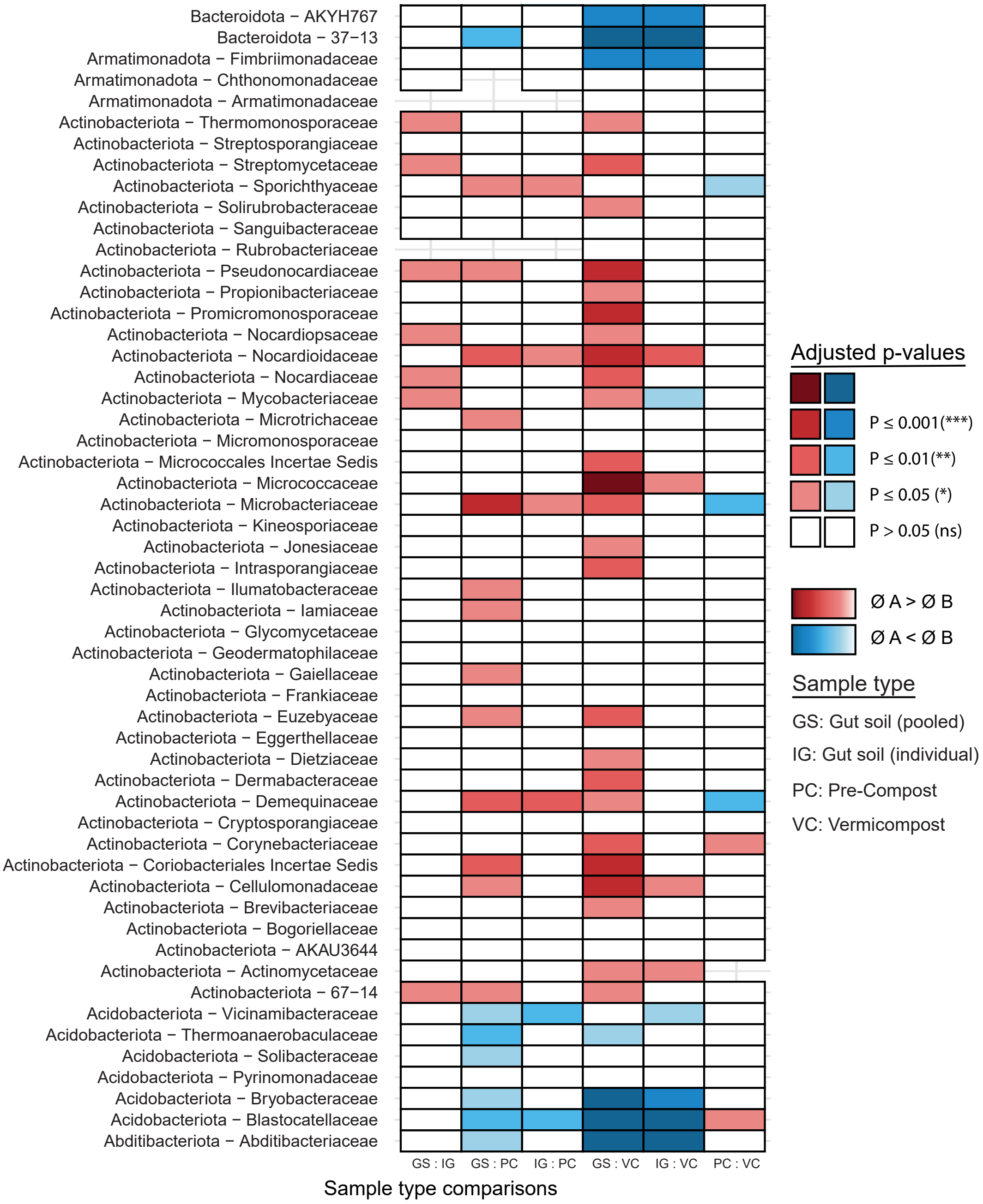

Figure S2. Pair wise comparisons of each sample type (GS), individual gut soil (IG), Pre-compost (PC) and Vermicompost (VC) for each family identified by Wilcoxon test with post Benjamini-Hochberg showing adjusted p-values for each comparison ($p > 0.05$ (ns), $p \leq 0.05$ (*), $p \leq 0.01$ (**), $p \leq 0.001$ (***), $p \leq 0.0001$ (****)). Blue color scale indicates that the mean of sample A is lower that in sample B. Red color scale indicates that the mean of sample A is greater than the mean of sample B.

Figure S3. Rarefied taxonomic relative abundance plots with notable PUFA producers outlined. (**A**) Prokaryote 16S rRNA rarefied to 3k. (**B**) Eukaryote 18S rRNA with Oligochaetes removed and rarefied to 1k.

A. Prokaryote 16S taxa

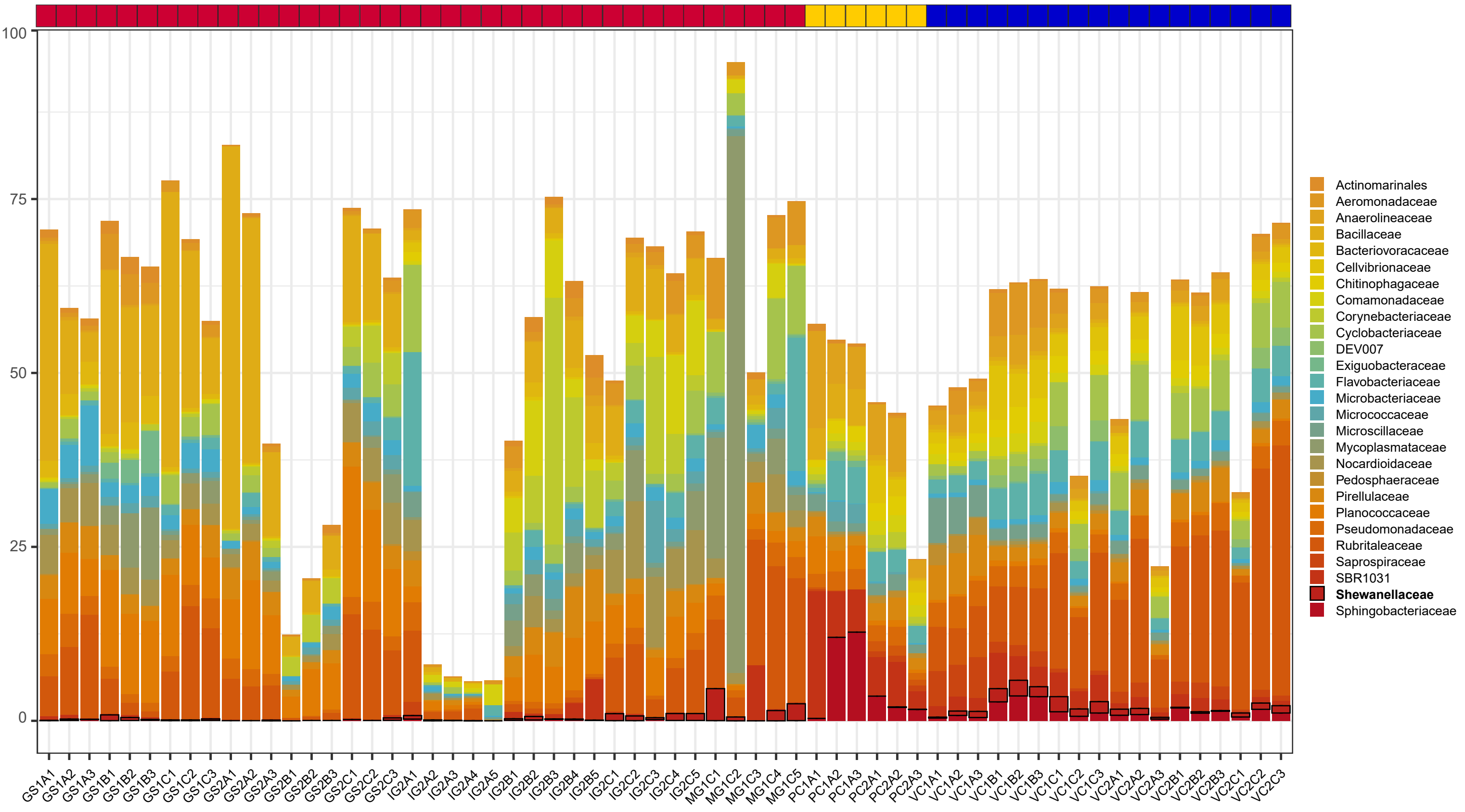

B. Eukaryote 18S taxa

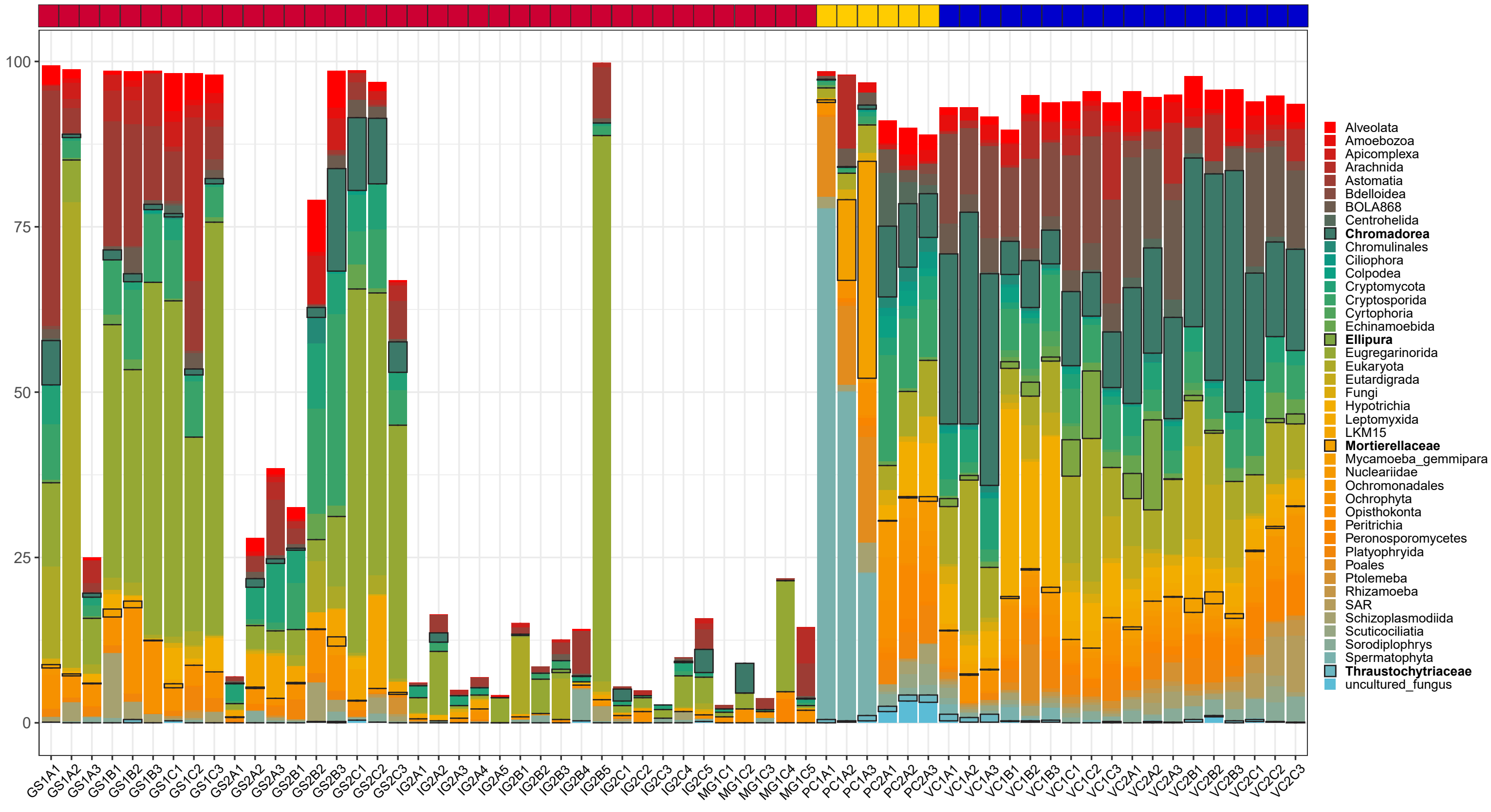

Figure S4. Phylogenetic distribution of PfaA-KS primer matches

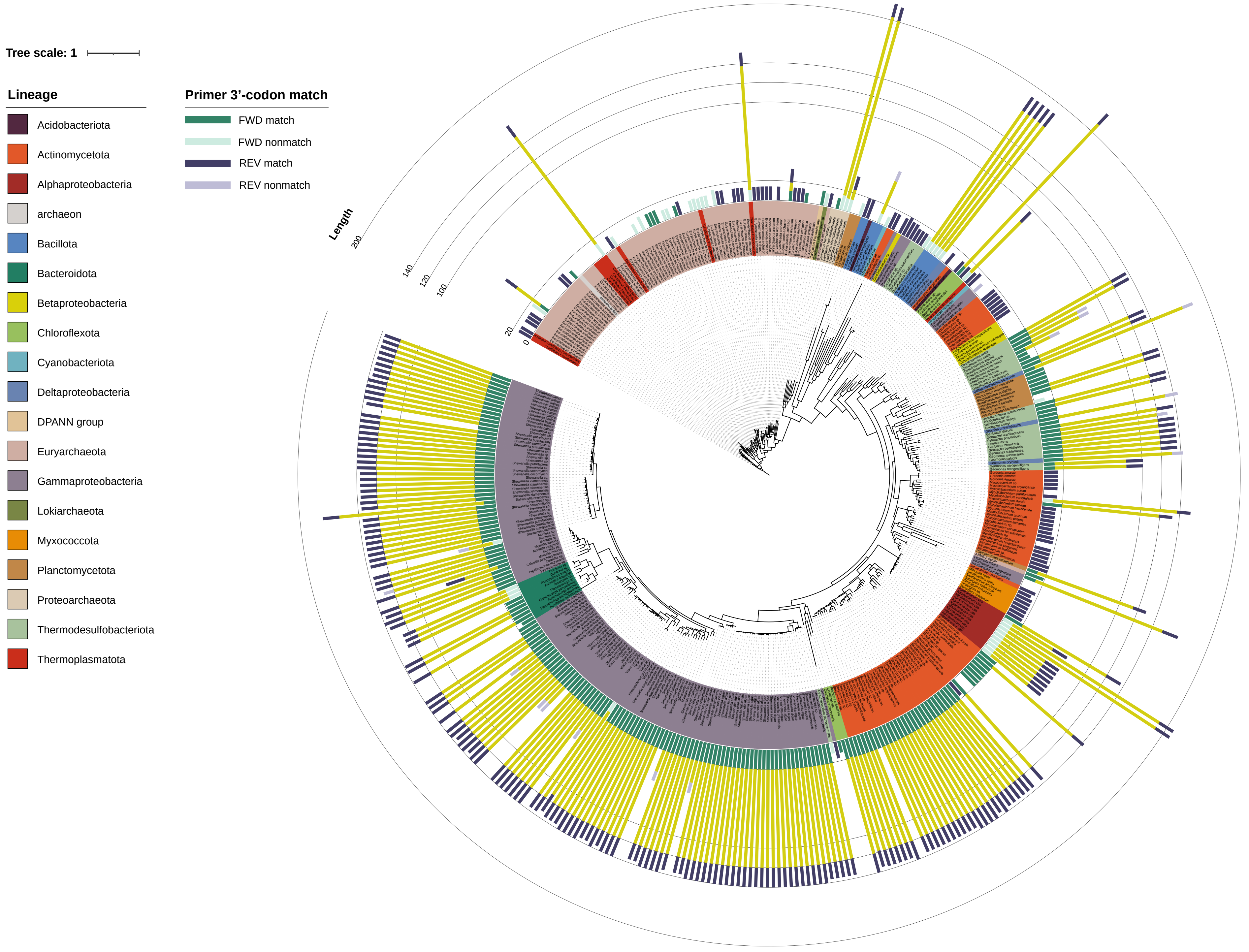

Figure S5. Associations between total PfaA-KS ASVs and metadata

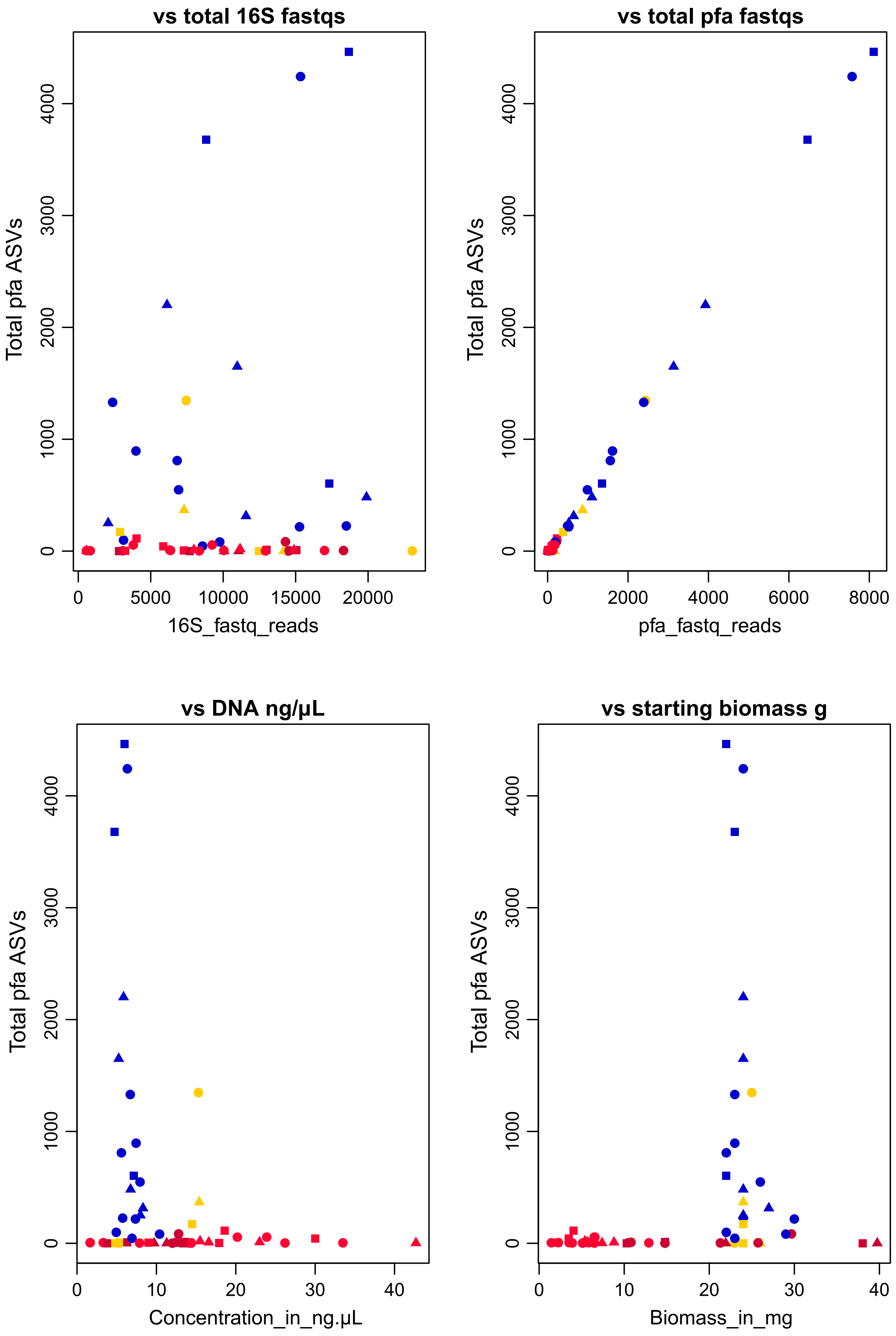

Figure S6. Abundance of PUFA taxa among 16S rRNA data

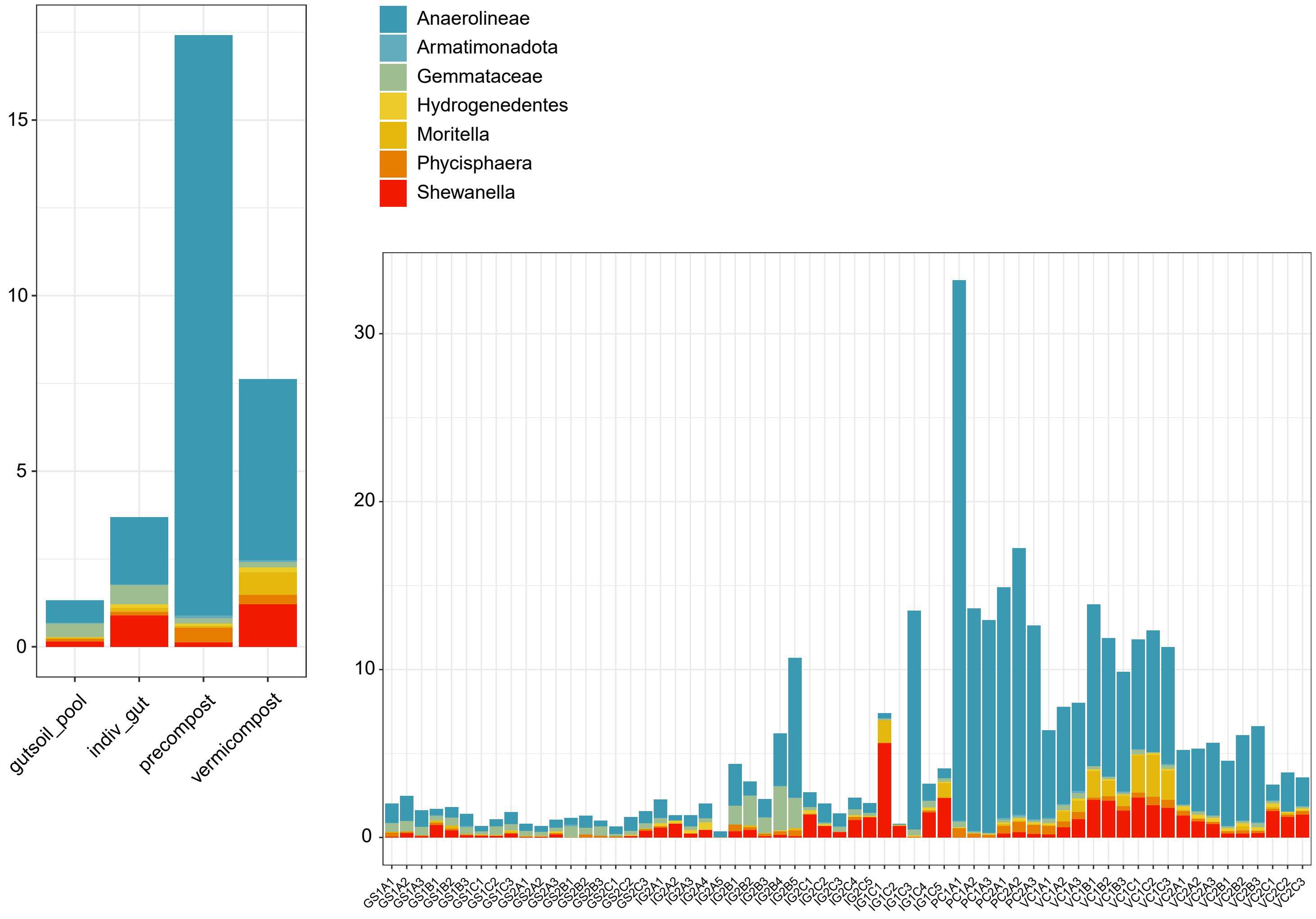

Figure S7. Mantel's test dotplot of **pfa** and **prok.pfa** datasets showing comparison of pairwise distances

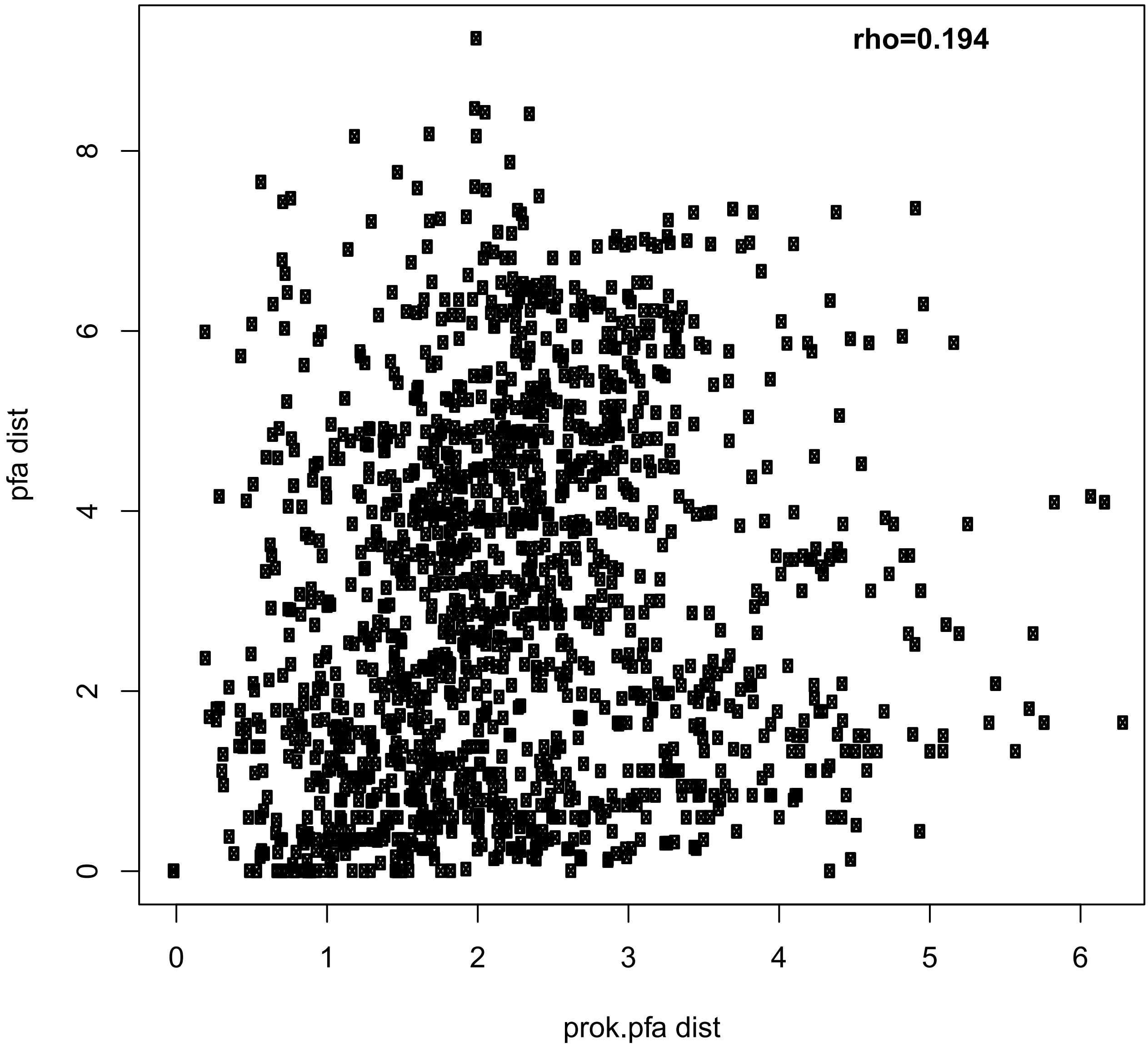

Figure S8. Linear associations between taxonomic abundance for pfa and prok.pfa datasets

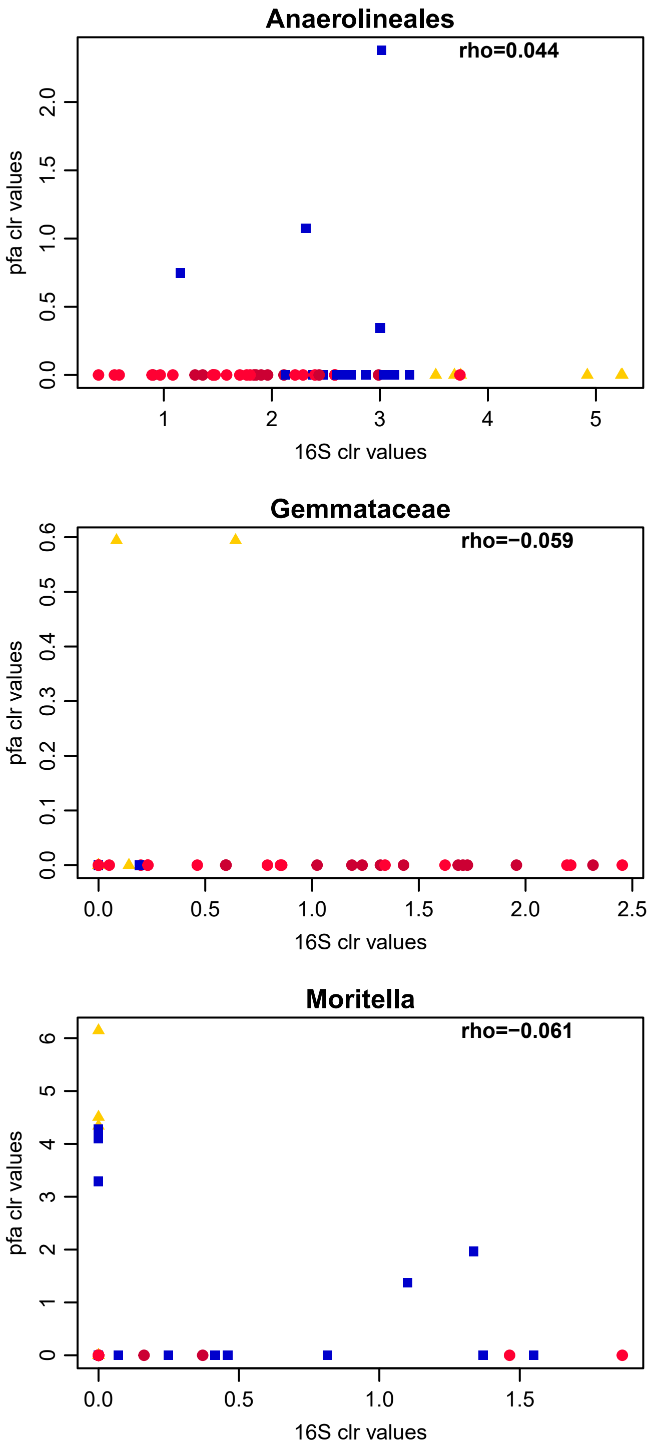

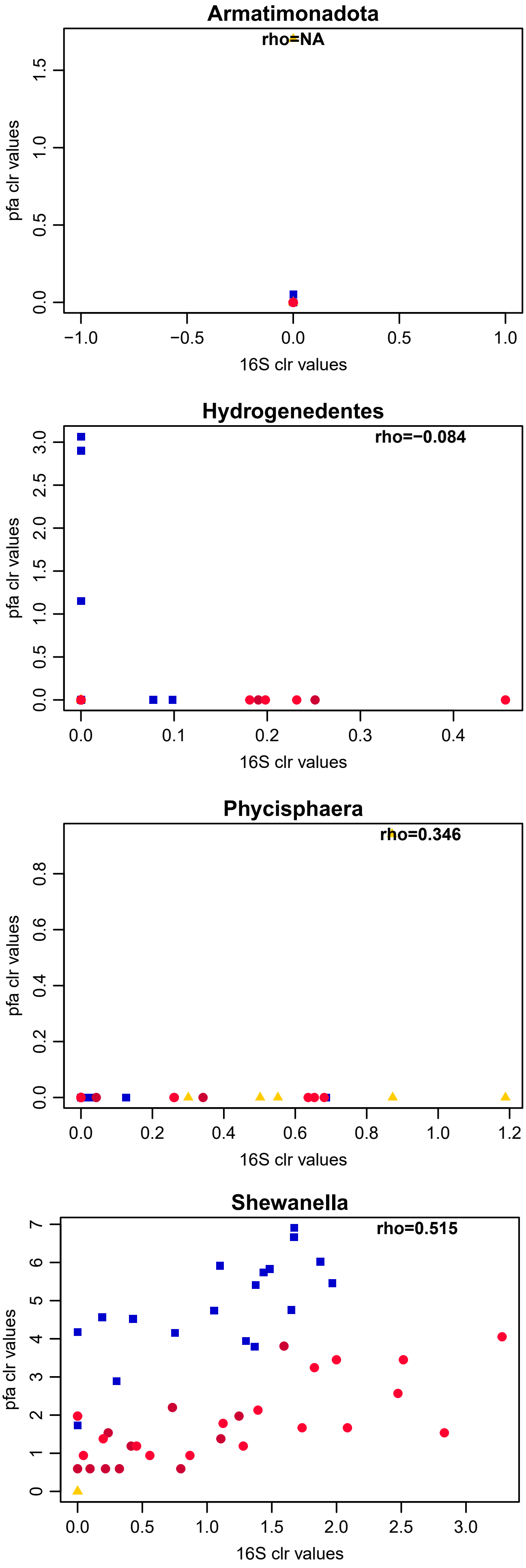

Figure S9. Distributions for pfa taxa in both datasets

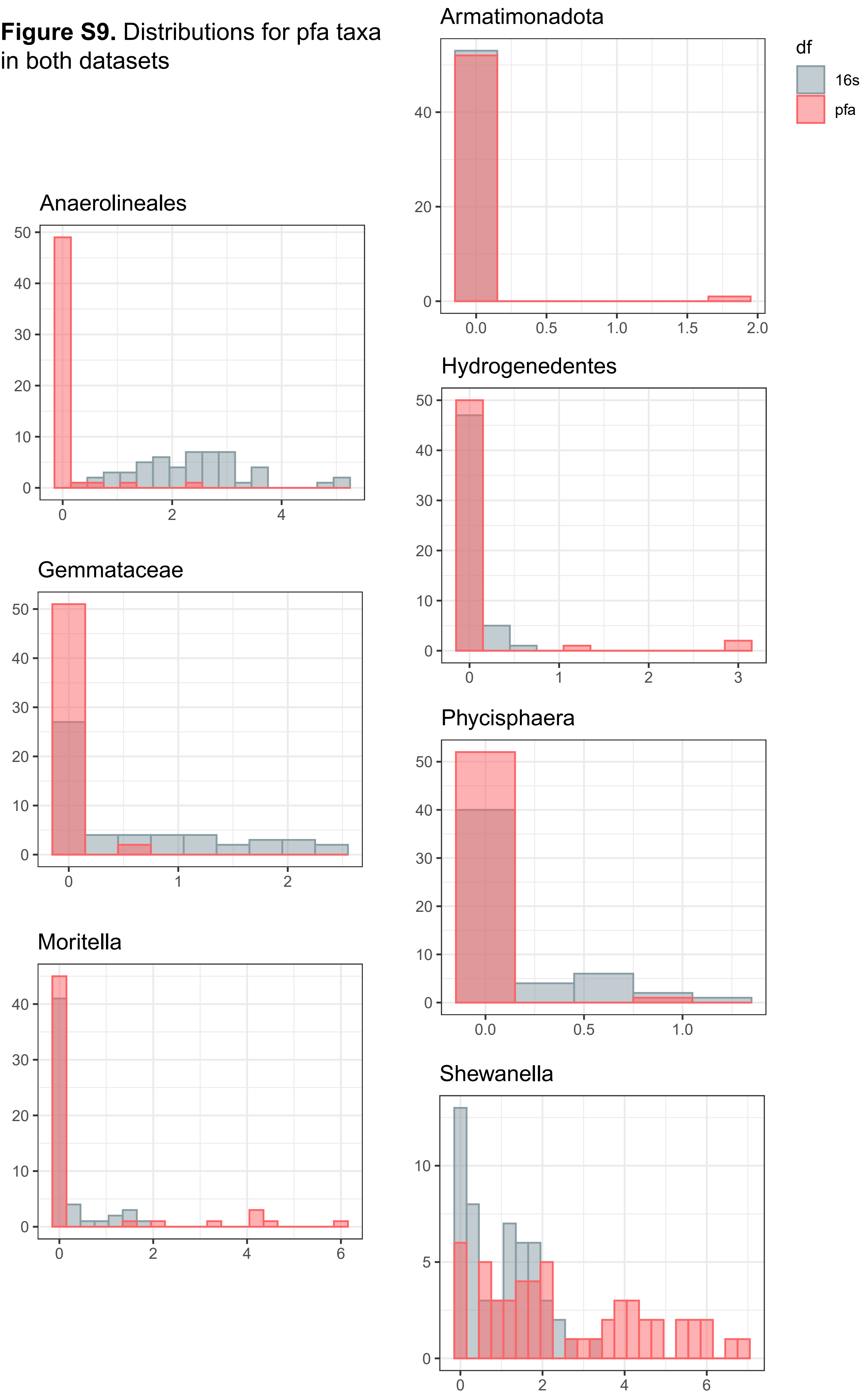

Figure S10. Effect size pairwise comparisons of the transformed abundances of pfa-taxa drawn from the prokaryote 16S dataset and compared across sample types

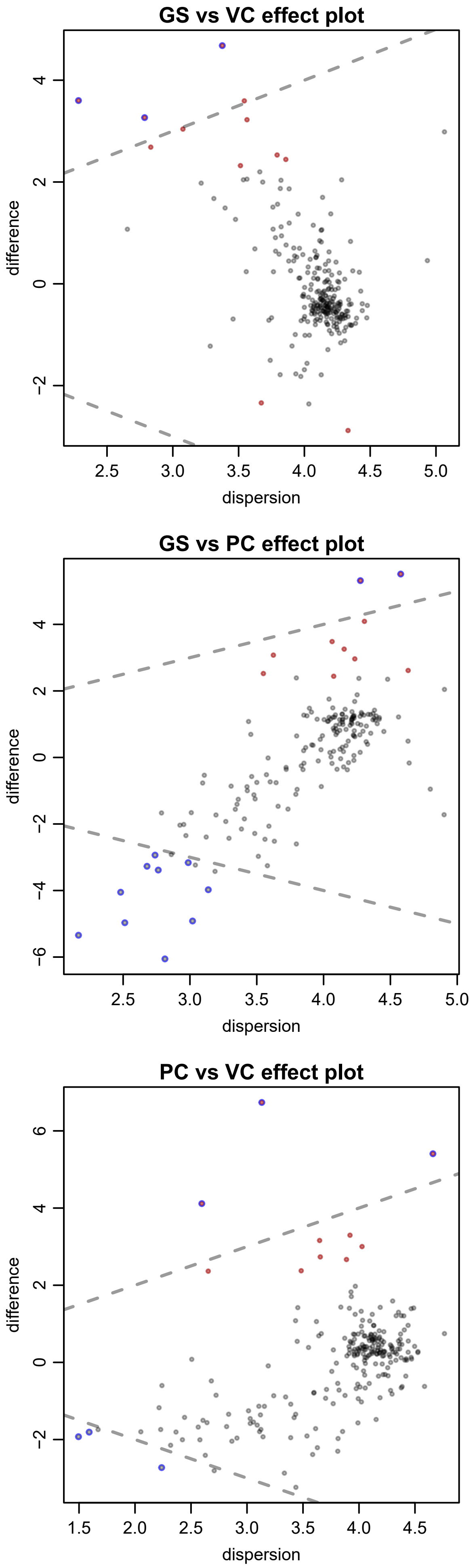

Supplement: Supplemental figures — Figures S1 to S10. [file aem.02069-24-s0001.pdf]
